# ^68^Ga-DOTA-Siglec-9 – a new imaging tool to detect synovitis

**DOI:** 10.1186/s13075-015-0826-8

**Published:** 2015-11-03

**Authors:** Helena Virtanen, Anu Autio, Riikka Siitonen, Heidi Liljenbäck, Tiina Saanijoki, Petteri Lankinen, Jussi Mäkilä, Meeri Käkelä, Jarmo Teuho, Nina Savisto, Kimmo Jaakkola, Sirpa Jalkanen, Anne Roivainen

**Affiliations:** Turku PET Centre, University of Turku and Turku University Hospital, Kiinamyllynkatu 4-8, Turku, FI-20521 Finland; Turku Center for Disease Modeling, University of Turku, Turku, Finland; Department of Orthopaedic Surgery and Traumatology, University of Turku, Turku, Finland; Department of Cell Biology and Anatomy, University of Turku, Turku, Finland; VTT Technical Research Centre of Finland, Medical Biotechnology, Turku, Finland; MediCity Research Laboratory, University of Turku, Turku, Finland

**Keywords:** Gallium-68, Inflammation, PET, Rabbit, Vascular adhesion protein-1

## Abstract

**Introduction:**

Vascular adhesion protein-1 (VAP-1) is an adhesion molecule, which upon inflammation is rapidly translocated from intracellular sources to the endothelial cell surface. We have recently discovered that sialic acid- binding immunoglobulin-like lectin 9 (Siglec-9) is a leukocyte ligand of VAP-1 and that ^68^Ga-labeled Siglec-9 motif peptide facilitates in vivo imaging of inflammation. This study evaluated the feasibility of ^68^Ga-DOTA-Siglec-9 positron emission tomography (PET) for the assessment of synovitis.

**Methods:**

Rabbits with synovial inflammation were injected with ^18^F-FDG or ^68^Ga-DOTA-Siglec-9 and studied by gamma counting and autoradiography. Certain rabbits were also examined with magnetic resonance imaging (MRI). After PET imaging, rabbits were intravenously administered with anti-VAP-1 antibody to evaluate luminal expression of VAP-1 by immunohistochemistry. Finally, binding of Siglec-9 peptide and VAP-1 positive vessels were evaluated by double staining of rheumatoid arthritis synovium.

**Results:**

Intra-articular injection of hemagglutinin induced mild synovial inflammation in rabbit knee with luminal expression of VAP-1. Synovitis was clearly visualized by ^68^Ga-DOTA-Siglec-9 PET in addition to ^18^F-FDG-PET and MRI. Compared with the ^18^F-FDG, the *ex vivo* inflamed-to-control synovium ratio of ^68^Ga-DOTA-Siglec-9 was similar (1.7 ± 0.4 vs. 1.5 ± 0.2, *P* = 0.32). Double staining revealed that Siglec-9 peptide binds to VAP-1 positive vessels in human rheumatoid synovium.

**Conclusion:**

Ga-DOTA-Siglec-9 PET tracer detected VAP-1 positive vasculature in the mild synovitis of rabbits comparable with ^18^F-FDG, suggesting its potential for in vivo imaging of synovial inflammation in patients with rheumatic diseases.

## Introduction

Rheumatoid arthritis (RA) is a chronic, progressive, inflammatory disease with local (joint) and systemic inflammatory manifestations. Early diagnosis is important for the purposes of starting effective disease-suppressing therapy before permanent damages occur. RA synovitis is characterized by local accumulation of lymphocytes, and synovial hyperplasia and angiogenesis [1]. Synovial neovascularization is an essential feature in the progression of RA as it allows the entry of circulating leukocytes, thereby exacerbating inflammation. It also provides nutrients to the hyperproliferative synovium [2]. Conventional radiography may detect anatomical changes, such as bone erosion and cartilage damage, but more sensitive imaging techniques are needed for the earlier diagnosis of RA by using, for example, ultrasound, magnetic resonance imaging (MRI), computed tomography (CT), single-photon emission computed tomography (SPECT) or positron emission tomography (PET). During the past two decades, several molecular imaging protocols have been developed for in vivo detection of inflammation, but, as yet, no imaging agent has been found that would have optimal characteristics for imaging of inflammation [3–5].

**Fig. 1:**
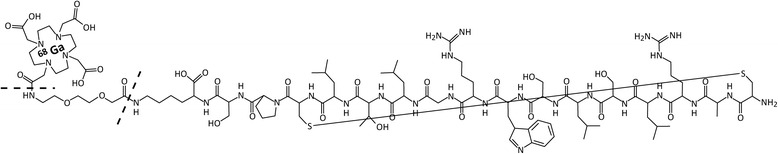
Molecular structure of ^68^Ga-DOTA-Siglec-9

Vascular adhesion protein-1 (VAP-1) is an endothelial cell molecule, which is involved in leukocyte trafficking from blood into the tissue. In normal conditions, the endothelial cell surface is practically VAP-1-negative. However, upon inflammation, VAP-1 is rapidly translocated from intracellular storage granules to the endothelial cell surface [6], where it contributes to leukocyte-endothelial adhesion in the early phases of inflammation. Although VAP-1 plays an important role in the early events of inflammation, its expression on the cell surfaces will remain constant for a longer time, if the inflammation continues. This makes VAP-1 a promising target for both anti-inflammatory therapy and in vivo imaging of inflammation. Indeed, several articles have been published, to date, concerning VAP-1 as a target for in vivo imaging [7–12]. Although leukocytes can bind to the endothelium via VAP-1, their counter-receptors were for a long time unknown [13]. Recently, it was found that sialic acid-binding immunoglobulin-like lectins 9 and 10 (Siglec-9 and Siglec-10) are leukocyte ligands of VAP-1 [14, 15]. In addition, we have previously demonstrated that Gallium-68-labeled Siglec-9 motif containing 1,4,7,10-tetraazacyclododecane-*N*,*N*′,*N*′′,*N*′′′-tetraacetic acid conjugated peptide (^68^Ga-DOTA-Siglec-9) can be used for PET imaging of inflammation and cancer [14, 16]. The ^68^Ga-DOTA-Siglec-9 (C_104_H_174_N_30_O_32_S_2_, molecular weight 2420.2 g/mol) is a cyclic CARLSLSWRGLTLCPSK peptide with disulfide-bridged cysteines, consisting of residues 283 − 297 from the Siglec-9. Additionally, the tracer has 8-amino-3,6-diooxaoctanoyl linker (polyethylene glycol derivative) between DOTA chelator and the peptide (Fig. 1).

In this study, the aim was to evaluate the feasibility of VAP-1 targeting ^68^Ga-DOTA-Siglec-9 peptide for the assessment of synovitis by PET imaging. For the purposes of comparison and validation of the rabbit model of mild synovitis, PET with glucose analog 2-deoxy-2-[^18^F]-fluoro-*D*-glucose (^18^F-FDG) was also performed.

## Methods

### Radiochemistry

^68^Ga was obtained from a ^68^Ge/^68^Ga generator (Eckert & Ziegler, Valencia, CA, USA) by elution with 0.1 M HCl. ^68^Ga eluate (0.5 mL, 280 − 360 MBq) was mixed with 2-[4-(2-hydroxyethyl)piperazin-1-yl]ethanesulfonic acid (HEPES, 120 mg) to give a pH of approximately 4.1. DOTA-Siglec-9 peptide (5 − 35 nmol, 12 − 85 μg, dissolved in deionized water to give a stock solution of 1 mM; Peptide Specialty Laboratories GmbH, Heidelberg, Germany) was added, and the reaction mixture was heated at 100 °C for 15 minutes. No further purification was performed. The radiochemical purity of ^68^Ga-DOTA-Siglec-9 was determined by radiodetector-coupled reversed-phase high-performance liquid chromatography (radio-HPLC) (Jupiter C18 column, 4.6 × 150 mm, 300 Å, 5 μm; Phenomenex, Torrance, CA, USA). The HPLC conditions were as follows: flow rate = 1 mL/minute; λ = 215 nm; A = 0.1 % trifluoroacetic acid (TFA)/water; B = 0.1 % TFA/acetonitrile; gradient: during 0 − 2 minutes 82 % A and 18 % B; during 2 − 11 minutes from 82 % A and 18 % B to 40 % A and 60 % B; during 11 − 15 minutes from 40 % A and 60 % B to 82 % A and 18 % B; during 15 − 20 minutes 82 % A and 18 % B. The radio-HPLC system consisted of LaChrom Instruments (Hitachi; Merck, Darmstadt, Germany) and of a Radiomatic 150TR radioisotope detector (Packard, Meriden, CT, USA). ^18^F-FDG was synthesized as described previously [17].

### *In vitro* stability of ^68^Ga-DOTA-Siglec-9

Tracer was incubated as such at room temperature for 4 h, or mixed with rabbit plasma and incubated at 37 °C for 1 h. At selected time points, aliquots were treated with acetonitrile (1:1, *v*/*v*) to precipitate the plasma proteins. After centrifugation (3 minutes, 3,900 × *g*), the supernatant was analyzed using radio-HPLC as described above except a larger Jupiter C18 column (10 × 250 mm, 300 Å, 5 μm; Phenomenex, Torrance, CA, USA) was used.

### Animal model and study design

All animal experiments were approved by the national Animal Experiment Board in Finland (ELLA) and the Regional State Administrative Agency for Southern Finland (ESAVI) and conducted in accordance with the European Union Directive. In order to induce knee joint synovitis in rabbits, fifteen male New Zealand White rabbits (weight 3.2 ± 0.6 kg) were intra-articularly injected with 80 μg phytohemagglutinin (Sigma-Aldrich, St Louis, MO, USA) in 200 μL sterile Roswell Park Memorial Institute (RPMI) medium (Gibco, Carlsbad CA, USA) [7]. Nine rabbits were used for model validation studies. Three rabbits were imaged with ^18^F-FDG at 8 h and four rabbits at 24 h after the induction of the inflammation. In two rabbits, synovitis was evaluated with gadolinium (Gd)-enhanced MRI performed at 24 h after phytohemagglutinin-induced inflammation. Seven rabbits were studied with ^68^Ga-DOTA-Siglec-9 peptide at 24 h after the induction of synovial inflammation. In addition to in vivo PET imaging, tracer uptake was evaluated by *ex vivo* gamma counting and digital autoradiography. In addition, the histology and luminal expression of VAP-1 in synovial tissues were studied.

### PET studies

For PET imaging, rabbits were anesthetized with medetomidine (Domitor® 0.1 mg/kg Orion Pharma, Espoo, Finland) and ketamine (Ketalar® 15 mg/kg, Pfizer, Dublin, Ireland), ear vein cannulated and intravenously (i.v.) administered with 49 ± 9 MBq of ^18^F-FDG or with MBq (1.6 ± 1.4 nmol, 4.0 ± 3.6 μg) of ^68^Ga-DOTA-Siglec-9 peptide. Animals were imaged with a High Resolution Research Tomograph (Siemens Medical Solutions, Knoxville, TN, USA), which is a dedicated brain/animal PET camera [18]. The 20-minute ^18^F-FDG PET acquisition started at 40 minutes after tracer injection, whereas the 30-minute ^68^Ga-DOTA-Siglec-9 PET started at the time of injection. The data acquired in a list mode were iteratively reconstructed with a 3-D ordered subsets expectation-maximization algorithm with 8 iterations, 16 subsets, and a 2-mm full-width at half-maximum post-filter into 4 × 300 s time frames for ^18^F-FDG and into 8 × 30 s, 6 × 60 s and 4 × 300 s time frames for ^68^Ga-DOTA-Siglec-9.

Quantitative analysis was performed by defining regions of interest (ROIs) on the inflamed knee, contralateral intact knee, femoral muscle and abdominal aorta (blood pool) using Carimas 2.8 software (Turku PET Centre, Turku, Finland; [19]). The average radioactivity concentration kBq/mL in the ROI was used for further analyses. The uptake was reported as a standardized uptake value (SUV), which was calculated as the radioactivity concentration of the ROI normalized with the injected radioactivity dose and animal weight. Radioactivity remaining in the cannula was compensated. Mean time-radioactivity curves extracted from dynamic PET images were used for presenting the kinetics of the ^68^Ga-DOTA-Siglec-9 uptake.

During the PET imaging, 10 minutes before being killed, the animals were i.v. injected with anti-VAP-1 antibody (BTT-1023 1 mg/kg, Biotie Therapies Corp., Turku, Finland). Rabbits were sacrificed and various tissue samples (adrenal gland, blood, contralateral control synovium, heart, inflamed synovium, intraperitoneal fat, kidney, liver, lung, lymph nodes, femoral muscle, skin, spleen and urine) were excised, weighed and measured for radioactivity using a gamma counter (1480 Wizard 3", PerkinElmer/Wallac, Turku, Finland). Results were expressed as SUV.

*Ex vivo* distribution of ^68^Ga-DOTA-Siglec-9 was studied in more detail with digital autoradiography. Inflamed and intact synovial tissue samples were frozen with dry ice, sectioned with cryomicrotome into 8 μm and 20 μm sections at –15 °C, thaw-mounted onto microscope slides, and the 20-μm sections were apposed to an imaging plate (Fuji Photo Film Co., Ltd, Tokyo, Japan). After an exposure time of 2.5 h, the imaging plates were scanned with the Fuji Analyzer BAS-5000 (Fuji Photo Film Co., Ltd, Tokyo, Japan; internal resolution of 25 μm) to produce digitalized images. The images were analyzed for count densities (photostimulated luminescence units (PSL)/mm^2^) using TINA version 2.10f software (Raytest Isotopenmessgeräte GmbH, Straubenhardt, Germany). ROIs were defined in accordance with hematoxylin and eosin staining. The radioactivity uptake was expressed as PSL/mm^2^ normalized for the injected radioactivity dose, animal weight and the radioactivity decay. The background count densities were subtracted from the image data. Several tissue sections were analyzed for each animal and the results are expressed as mean ± SD values.

### Histology and immunohistochemistry

After autoradiography, the 20-μm synovium cryosections were stained with hematoxylin and eosin, and studied for histology under a light microscope. Detection of luminal VAP-1 by i.v. administered anti-VAP-1 antibody was studied with immunohistochemical staining, applying fluorescently labeled secondary antibody on 8-μm cryosections [10].

### *In situ* binding of Siglec-9 peptide in rheumatoid synovium

Frozen sections of human inflamed synovia were first incubated for 30 minutes with biotinylated Siglec-9 peptide (20 micrograms/mL in Dulbecco’s phosphate-buffered saline (PBS) containing magnesium and calcium (Sigma) followed by streptavidin-phycoerythrin. After the washes in PBS, the sections were incubated with fluorescein-conjugated anti-VAP-1 antibody (JG2.10) 10 micrograms/mL for 30 minutes. Staining without the peptide served as a negative control. The sections were analyzed using the Olympus 60X fluorescence microscope.

### MRI

Imaging was performed with a Philips Ingenuity TF PET/MR (Philips Healthcare, Cleveland, OH, USA), which consists of a 3.0 T Achieva 3 T X-series MRI and Gemini TF PET scanner [20]. The joints were examined by using a 2-element Philips SENSE Flex-S coil with a diameter of 11 cm. The coil was positioned in parallel with each knee, allowing simultaneous acquisition of images of both knees. All acquisitions were performed with parallel acquisition (SENSE) enabled, with a SENSE factor of 2.

The MRI protocol consisted of transverse and coronal T2 and proton density weighted (PDW) acquisitions of the knees. The T2 weighted acquisition was a turbo spin echo (TSE) sequence, with repetition time (TR) of 3,000 ms and echo time (TE) of 100 ms. The PDW images were acquired with a TSE sequence with a TR of 3,000 ms and TE of 30 ms. Both acquisitions were performed with a slice thickness of 1 mm and field- of- view (FOV) of 211 × 211 mm^2^. Transverse and coronal T1 weighted images were acquired before, immediately after and 10 minutes after the injection of Gd-contrast agent (Dotarem® 0.1 mmol/kg, Guerbet, Roissy Charles-de-Gaulle Cedex, France). The T1 acquisition consisted of a TSE sequence with a TR of 572 ms and TE of 20 ms. The slice thickness and FOV were 0.8 mm and 209 × 209 mm, respectively.

### Statistical analysis

All results are expressed as mean ± SD values. The Student *t* test was used to compare numerical variables between two groups. Statistical dependence between two variables was tested with Pearson correlation. *P* values less than 0.05 were considered statistically significant. Statistical analyses were conducted using Origin version 7.5 software (OriginLab Corp., Northampton, UK).

## Results

### Radiochemistry and *in vitro* stability of ^68^Ga-DOTA-Siglec-9

**Fig. 2:**
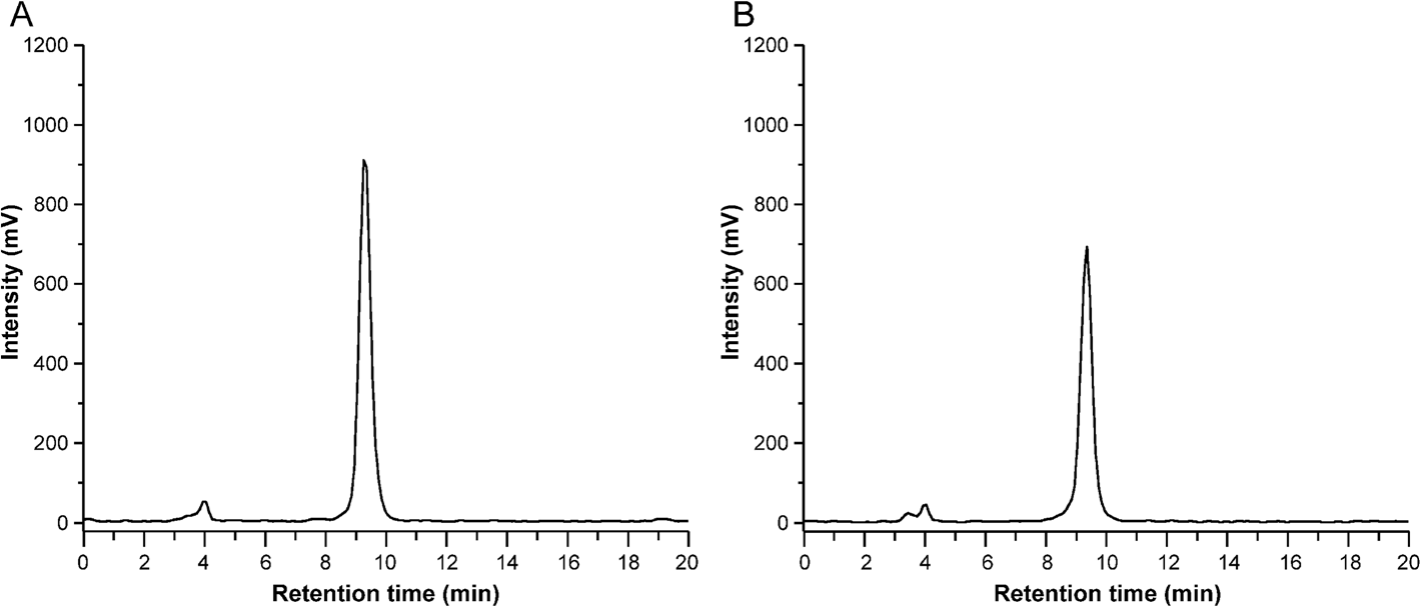
Representative radio-HPLC chromatograms of intact ^68^Ga-DOTA-Siglec-9 (retention time 9.25 minutes) (**a**) and rabbit plasma *in vitro* incubated with the tracer for 1 h at 37 °C (retention time 9.35 minutes) (**b**)

The radioactivity concentration, specific radioactivity and radiochemical purity of ^68^Ga-DOTA-Siglec-9 were 457 MBq/mL, 41 ± 18 GBq/μmol and 96 ± 2.8 %, respectively. After incubation at room temperature for 4 h as such or at 37 °C for 1 h in rabbit plasma, the radioactivity associated with intact radiotracer was 97 ± 0.5 % (n = 3) and 94 ± 1.0 % (n = 2), respectively. These results indicate that ^68^Ga-DOTA-Siglec-9 was highly stable *in vitro*. Representative radio-HPLC chromatograms are shown in Fig. 2.

### Rabbit model of synovitis and initial ^18^F-FDG PET and MRI findings

**Fig. 3:**
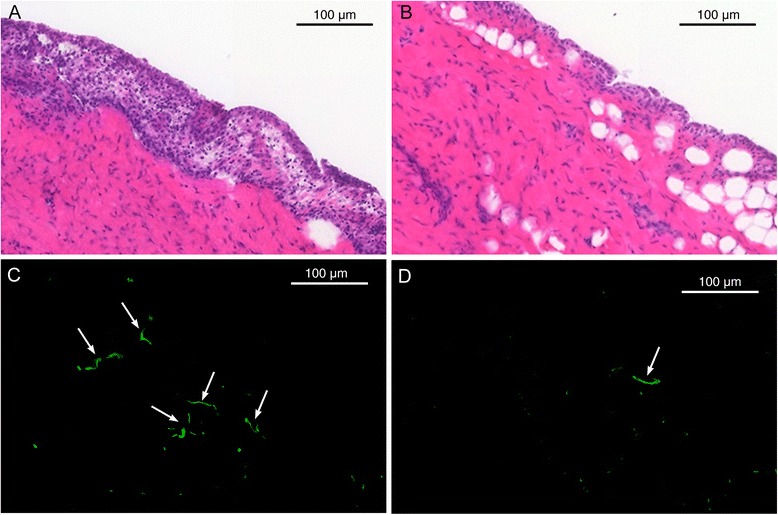
Intra-articular injection of phytohemagglutinin induced a mild inflammation and luminal expression of vascular adhesion protein 1 (VAP-1) in rabbit knee synovium. Hematoxylin-eosin staining of inflamed (**a**) and control (**b**) synovial tissue. Fluorescence-based anti-VAP-1 immunohistochemistry of inflamed (**c**) and contralateral intact (**d**) synovium. *Arrows* indicate VAP-1-positive vessels. *Scale bar* is 100 μm

Intra-articular injection of phytohemagglutinin induced a mild synovial inflammation in rabbit knees. Swelling, redness and warmness of the joint were observed by palpation as early as 8 h after induction. Hematoxylin- eosin staining demonstrated thickening of synovial membrane with infiltration of inflammatory cells when compared to the contralateral intact synovium (Figs. 3a and b). Intravenous injection of anti-VAP-1 antibody followed by immunohistochemical staining with a fluorescent secondary antibody, demonstrated luminal expression of VAP-1 in the inflamed synovium (Fig. 3c). Only occasional VAP-1-positive vessels were found in the control synovium, which probably reflects systemic response to the chemically induced inflammation (Fig. 3d).

**Fig. 4:**
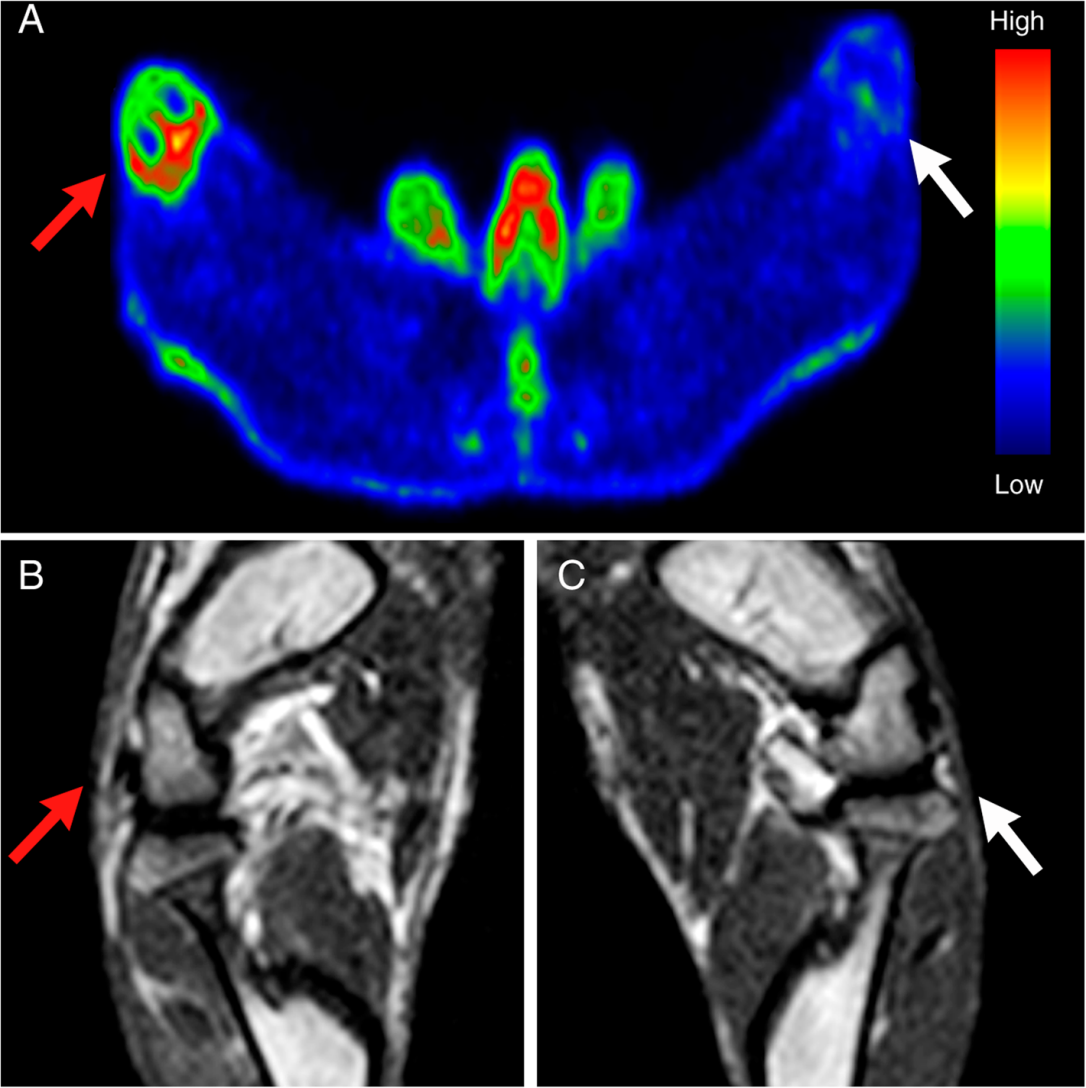
Detection of mild synovitis with ^18^F-FDG positron emission tomography (PET) and gadolinium (Gd)-enhanced magnetic resonance imaging (MRI). **a** Representative transaxial ^18^F-FDG PET image of rabbit knees. Phytohemagglutinin-induced inflammation had a standardized uptake value (SUV) of 1.20 and the healthy knee had an SUV of 0.75. Representative coronal Gd-enhanced T1-weighted MR images of an inflamed knee (**b**) and a control knee (**c**). *Red arrows* indicate the inflamed synovium and *white arrows* the control synovium

Synovial inflammation was clearly visualized in vivo with ^18^F-FDG PET (Fig. 4a) and Gd-enhanced T1-weighted MRI (Figs. 4b and c). With ^18^F-FDG PET, the inflamed-to-control joint ratios were 1.5 ± 0.4 (*P* = 0.095) and 1.6 ± 0.2 (*P* = 0.0020) at 8 h and 24 h after phytohemagglutinin-induced inflammation, respectively. The difference between the ratios at 8 h and 24 h was not statistically significant (*P* = 0.14). With *ex vivo* gamma counting, the inflamed-to-control synovium ratios were 1.3 ± 0.2 (*P* = 0.059) and 1.5 ± 0.2 (*P* = 0.014) at 8 h and 24 h, respectively. Also with these measures, the differences between the 8-h and 24-h groups were not statistically significant (*P* = 0.36). Thus, the in vivo and *ex vivo*^18^F-FDG measures correlated well (*r* = 0.84, *P* <0.001). However, as the target-to-background ratios were slightly higher at 24 h after induction of inflammation, this time point was selected for ^68^Ga-DOTA-Siglec-9 studies.

### ^68^Ga-DOTA-Siglec-9 detects mild synovitis in vivo

**Fig. 5:**
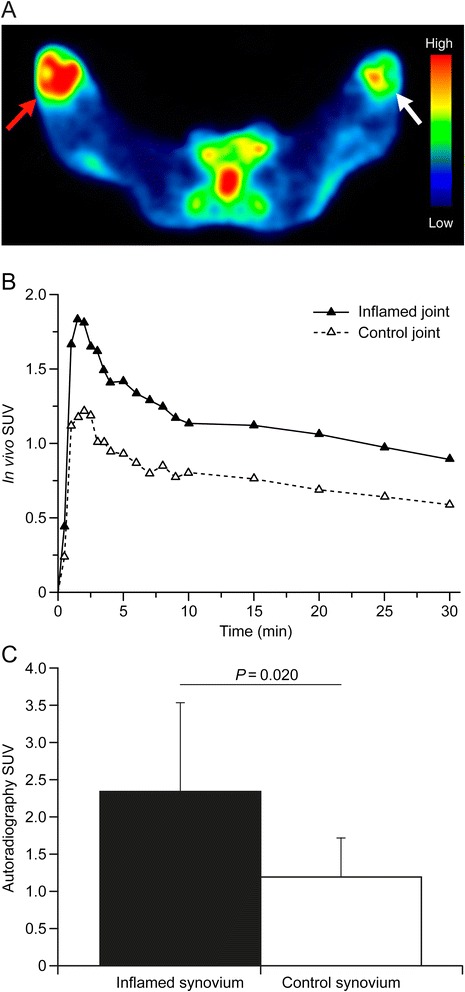
^68^Ga-DOTA-Siglec-9 detects mild synovitis in rabbits. **a** Representative transaxial ^68^Ga-DOTA-Siglec-9 PET image of rabbit knees. *Red arrow* indicates the phytohemagglutinin-induced inflammation (standardized uptake value (*SUV*) = 1.30) and *white arrow* the healthy knee (SUV = 0.86). **b** Corresponding radioactivity concentration as a function of time. **c** ^68^Ga-DOTA-Siglec-9 uptake assessed by digital autoradiography of excised synovial tissue samples. Values are mean ± SD

^68^Ga-DOTA-Siglec-9 PET was able to visualize mild synovitis in rabbit knee (Fig. 5a). The peak of ^68^Ga-DOTA-Siglec-9 uptake was observed approximately 2 minutes after the i.v. bolus injection, followed by a decrease and plateau after 10 minutes (Fig. 5b). According to intra-animal comparison, the inflamed-to-control joint ratio was on average 1.2 ± 0.14 (*P* <0.001) at 10 − 30 minutes after the tracer injection. *Ex vivo* biodistribution measurements performed at 30 minutes after the injection of ^68^Ga-DOTA-Siglec-9, as shown in Table 1, verified the in vivo PET results. The correlation between in vivo and *ex vivo* PET results was good (*r* = 0.72, *P* <0.001). Digital autoradiography results of ^68^Ga-DOTA-Siglec-9 distribution were in line with in vivo and *ex vivo* PET results; the inflamed-to-control synovium ratio was 2.3 ± 1.2 (*P* = 0.020, Fig. 5c).

**Table 1:** Biodistribution of ^68^Ga-DOTA-Siglec-9 in rabbits with mild synovitis

Tissue	Standardized uptake value
Adrenal gland	0.4 ± 0.1
Blood	1.8 ± 0.6
Control synovium	0.8 ± 0.2
Heart	0.5 ± 0.1
Inflamed synovium	1.3 ± 0.2
Intraperitoneal fat	0.2 ± 0.1
Kidney	4.3 ± 1.0
Liver	1.4 ± 0.7
Lung	0.8 ± 0.2
Lymph nodes	0.8 ± 0.2
Muscle	0.2 ± 0.1
Skin	0.9 ± 0.2
Spleen	3.1 ± 1.9
Urine	42 ± 35

Results are expressed as mean ± SD and represent *ex vivo* gamma counting of excised tissue samples obtained 30 minutes after tracer injection

**Fig. 6:**
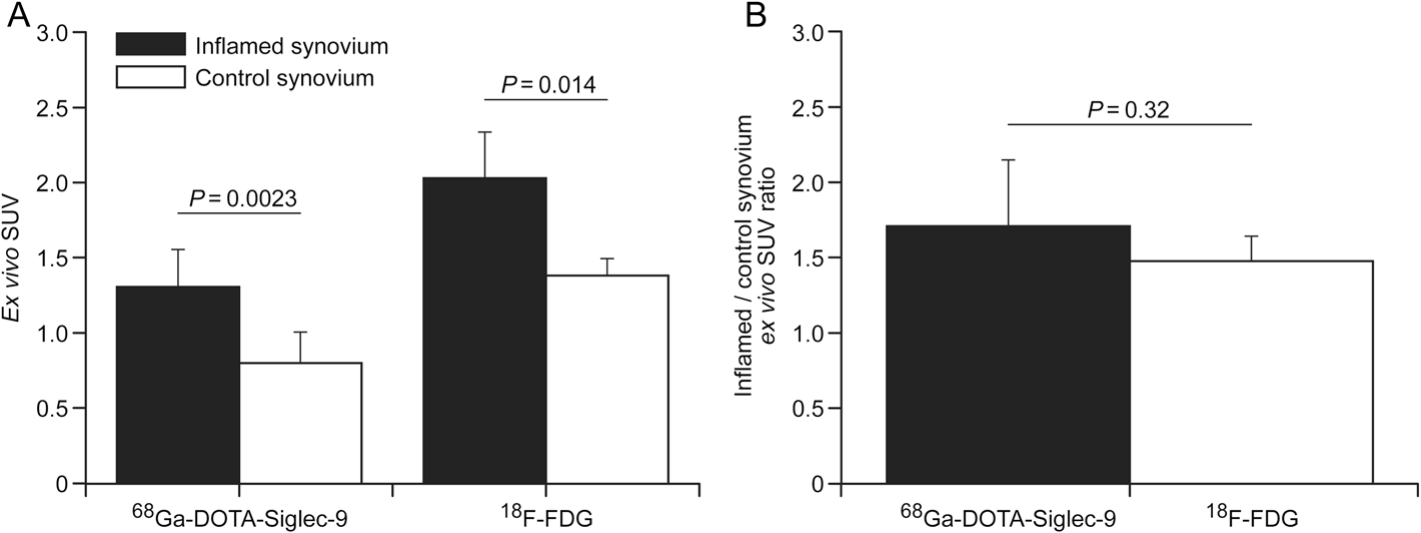
Comparison of ^68^Ga-DOTA-Siglec-9 and ^18^F-FDG for the detection of synovial inflammation in a rabbit model. Tracer uptakes are expressed as *ex vivo* standardized uptake value (*SUV*) (**a**) and *ex vivo* SUV ratios (**b**). Values are mean ± SD

The uptakes of ^68^Ga-DOTA-Siglec-9 and ^18^F-FDG were comparable in the inflamed synovium as compared to the control synovium (Fig. 6). The *ex* *vivo*-measured ^68^Ga-DOTA-Siglec-9 uptake ratio between the inflamed synovium and the intact contralateral synovium (1.7 ± 0.4, *P* = 0.0035) was higher as compared to ^18^F-FDG, but the difference was not statistically significant (*P* = 0.32, Fig. 6b).

### Siglec-9 peptide binds to VAP-1 positive vessels in rheumatoid synovium

**Fig. 7:**
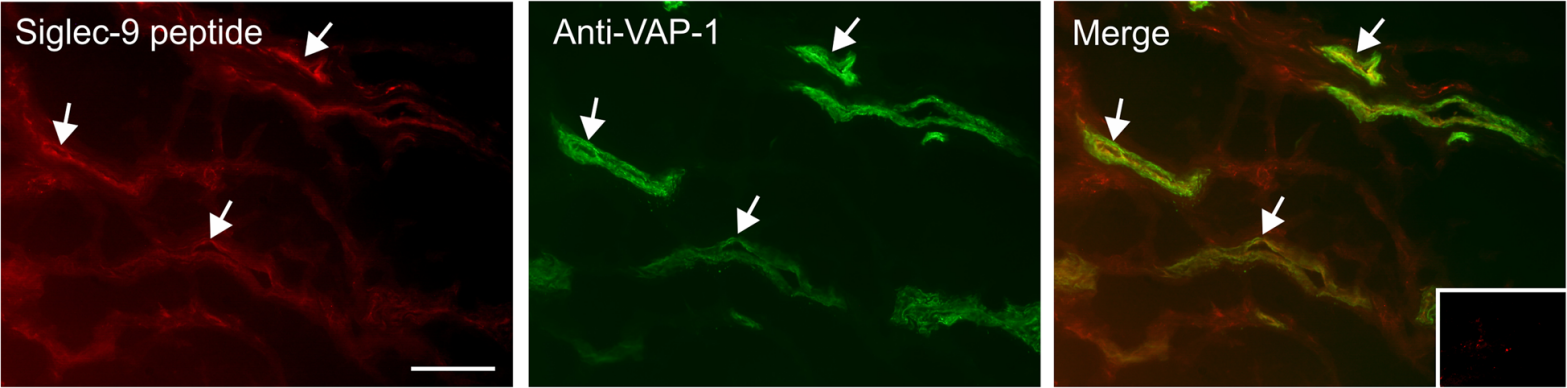
Siglec-9 peptide binds to vascular adhesion protein 1 (*VAP-1*)-positive vessels in human inflamed synovium. *Left panel*, binding of biotinylated Siglec-9 peptide (*red*). *Middle panel*, VAP-1-positive vessels detected by fluorescein-conjugated anti-VAP-1 monoclonal antibody (*green*). *Right panel*: merge. *Inset*, negative control staining. *Arrows* indicate some double-positive vessels. *Scale bar* is 100 μm

To get information about the potential of using Siglec-9 peptide in imaging of patients suffering from arthritis, we performed immunohistochemical double staining with biotinylated Siglec-9 peptide and anti-VAP-1 antibody using sections of synovial tissues affected by rheumatoid arthritis. These stains confirmed specific binding of the peptide to the VAP-1-positive vessels (Fig. 7).

## Discussion

The purpose of this study was to explore the feasibility of a novel VAP-1 targeting tracer, ^68^Ga-DOTA-Siglec-9 peptide, for the assessment of acute synovitis in a rabbit model in comparison with ^18^F-FDG. Our results revealed that ^68^Ga-DOTA-Siglec-9 PET was able to detect mild synovial inflammation induced by phytohemagglutinin and the tracer uptake was comparable with ^18^F-FDG. Subsequently, immunohistochemical double staining with biotinylated Siglec-9 peptide verified binding to VAP-1-positive vessels in rheumatoid synovium. This suggests the tracer’s potential for imaging of patients with RA or other types of arthritis.

The most commonly used PET tracer, ^18^F-FDG, accumulates at the sites of inflammation but it is not an inflammation-specific tracer. The uptake of ^18^F-FDG mostly takes place in the metabolically active cells and therefore ^18^F-FDG is used to detect a variety of conditions such as malignant and benign neoplasms and fractures, and infectious and inflammatory conditions [21, 22]. ^18^F-FDG PET imaging facilitates the quantification of metabolic activity of synovial cells in patients with RA or other types of arthritis. In RA synovial inflammation, the glucose utilization and thus, ^18^F-FDG accumulation, are increased in the proliferating synovial fibroblasts and activated macrophages and other inflammatory cells. One of the characteristics of RA is hypoxia, which also can enhance the uptake of ^18^F-FDG by both macrophages and fibroblasts [23].

Several radiopharmaceuticals have been developed and tested for quantitative imaging of arthritic synovitis. Of PET tracers, ^11^C-choline [24] and ^11^C-(*R*)-PK11195 [25] have shown the most promising results in clinical studies. However, both of these tracers have very rapid in vivo radiometabolism and carry a short-lived ^11^C radionuclide (T_1/2_ 20.4 minutes), which limits their use. In addition to the prototypic 18 kDa translocator protein (TSPO) ligand ^11^C-(*R*)-PK11195, numerous other macrophage-targeting tracers are in the preclinical evaluation phase [26, 27]. Most of them are labeled with ^18^F or ^68^Ga rather than ^11^C. We are currently the only research team developing VAP-1-targeting imaging agents. Some other leukocyte homing-associated molecules, for example, E-selectin and intercellular adhesion molecule-1 (ICAM-1), are also being investigated as targets for in vivo imaging of inflammation [28, 29].

In general, leukocyte trafficking to the sites of inflammation is mediated by a multistep adhesion cascade, where VAP-1 mediates leukocyte rolling and might also contribute to the transmigration [30]. Every step in the extravasation cascade is a precondition to the next, which also theoretically provides a multitude of potential therapeutic targets. However, in practice, the targeting of the homing-associated molecules has not proven very successful in clinical settings and, to date, very few therapeutic agents, such as anti-α_1_β_1_ and anti- α_4_β_1_ integrin antibodies, have reached the market [31]. So far, the clinical trials targeting VAP-1 have been promising in RA [32] and VAP-1-targeting PET imaging could contribute to early diagnosis of RA.

Siglec-9 is a leukocyte ligand of VAP-1 [15]. A synthetic peptide containing the Siglec-9 motif can be labeled with radionuclides, and it will bind to VAP-1 translocated to the endothelial cell surface upon inflammation. Early detection is essential in order to treat a disease and prevent subsequent harmful consequences. For example, RA is a risk factor for life-threatening cardiovascular diseases like atherosclerosis, which is a chronic progressive form of vascular inflammation [33, 34]. Instant treatment, which reduces inflammation in RA in many cases, is the primary goal of intervention. CT, MRI and ultrasound gives only limited information about the processes occurring prior to structural changes. Molecular PET imaging can provide more sensitive and quantitative information about the biochemical processes and status of the disease before and after treatment, which is useful for the evaluation of the efficacy of novel therapies. Therefore, imaging with ^68^Ga-DOTA-Siglec-9 could identify those patients having prominent upregulation of VAP-1 and thus, would potentially benefit from anti-VAP-1 antibody treatment. In this context, it should be remembered that ^68^Ga-DOTA-Siglec-9 binds to the enzymatic groove of VAP-1, leaving the antibody-binding site unoccupied and therapeutically accessible. Although ^68^Ga-DOTA-Siglec-9 detected mild rabbit synovitis comparably to ^18^F-FDG, it remains to be studied which one performs better for the evaluation of efficacy of anti-inflammatory treatment in man.

Many experimental arthritis models have been described, for example, in mice, rats, rabbits and monkeys [35–37]. The rationale for choosing the rabbit model of arthritis in the current study was the fact that the rabbit joints are better sized for in vivo PET imaging than the joints of mice or rats. Furthermore, it has been reported that rabbit VAP-1 is sufficiently homologous with human VAP-1 and can be detected with some of the existing antibodies against human VAP-1 [12, 38]. Importantly, VAP-1 is absent from all leukocytes, thus facilitating its detection on inflamed endothelium after i.v. injection of an imaging agent [6, 38].

For model validation in the present study, seven rabbits were evaluated with ^18^F-FDG PET at 8 h or 24 h after the phytohemagglutinin injection. The amount of phytohemagglutinin was based on earlier studies of VAP-1 in dogs and pigs [7]. Phytohemagglutinin is a toxic lectin, which is derived from plants; for example, red kidney beans (*Phaseolus vulgaris*) contain a large amount of phytohemagglutinin. In the current rabbit study, the common features of inflammation, such as warmness, redness and swelling, were seen as early as 8 h after induction of inflammation, and were further supported by the morphological difference between the inflamed and control synovial tissues. The ^18^F-FDG uptake in the inflamed knee was significantly higher than in the intact contralateral knee at 24 h after the induction of inflammation, but not yet at 8 h. We therefore decided that further studies with ^68^Ga-DOTA-Siglec-9 would be performed using 24-h phytohemagglutinin induction of synovial inflammation.

Previously, using the same rabbit model, we have demonstrated the imaging of synovial inflammation with ^124^I-labeled fully human anti-VAP-1 antibody, ^124^I-BTT-1023. For comparison, the *ex-vivo*-measured inflamed-to-control synovium SUV ratios of ^68^Ga-DOTA-Siglec-9 (1.7 ± 0.44) are in line with those of ^124^I-BTT-1023 (1.7 ± 0.5). In our previous study, we found VAP-1-positive vessels even in the intact joint in this animal model [12]. We consider this finding to be due to a systemic response to inflammation, which is caused by the chemical used for the induction of inflammation. In humans, vessels expressing VAP-1 on their surface have not been found in healthy synovial tissue. Phytohemagglutinin induced a mild synovitis and the level of VAP-1 expression was lower when compared to that of the patients with RA. However, the i.v. injection of anti-VAP-1 antibody, followed by immunofluorescence staining with a secondary antibody, verified that VAP-1 was translocated onto the endothelial cell surface.

## Conclusions

^68^Ga-DOTA-Siglec-9 PET tracer detected VAP-1-positive vasculature in the mild synovitis of rabbits comparable with ^18^F-FDG. These findings suggest that ^68^Ga-DOTA-Siglec-9 peptide is already a potential tracer for in vivo imaging of synovial inflammation at the early stages of the disease, for example, in patients with RA.

## Abbreviations

CT: Computed tomography
^18^F-FDG: 2-Deoxy-2-[fluorine-18]fluoro-*D*-glucose
FOV: Field-of-view
Gd: Gadolinium
HEPES: 2-[4-(2-Hydroxyethyl)piperazin-1-yl]ethanesulfonic acid
HPLC: High-performance liquid chromatography
i.v.: Intravenously
MRI: Magnetic resonance imaging
PBS: Phosphate-buffered saline
PDW: Proton density weighted
PET: Positron emission tomography
PSL/mm^2^: Photostimulated luminescence per square millimeter
RA: Rheumatoid arthritis
ROI: Region of interest
Siglec-9: Sialic acid- binding immunoglobulin-like lectin 9
SPECT: Single-photon emission computed tomography
SUV: Standardized uptake value
TE: Echo time
TFA: Trifluoroacetic acid
TR: Repetition time
TSE: Turbo spin echo
VAP-1: Vascular adhesion protein 1
DOTA: 1,4,7,10-tetraazacyclododecane-*N,N′,N′′,N′′′*-tetraacetic acid
